# Abnormally located SSEA1+/SOX9+ endometrial epithelial cells with a basalis-like phenotype in the eutopic functionalis layer may play a role in the pathogenesis of endometriosis

**DOI:** 10.1093/humrep/dey336

**Published:** 2018-11-29

**Authors:** D K Hapangama, J Drury, L Da Silva, H Al-Lamee, A Earp, A J Valentijn, D P Edirisinghe, P A Murray, A T Fazleabas, C E Gargett

**Affiliations:** 1 Department of Women’s and Children’s Health, Institute of Translational Medicine, University of Liverpool, Liverpool, UK; 2 Liverpool Women’s Hospital NHS Foundation Trust, Liverpool, UK; 3 Institute of Translational Medicine, University of Liverpool, Liverpool, UK; 4 Department of Obstetrics, Gynaecology and Reproductive Biology, College of Human Medicine, Michigan State University, Grand Rapids, MI, USA; 5 Department of Obstetrics and Gynaecology, Monash University, Clayton, Australia; 6 The Ritchie Centre, Hudson Institute of Medical Research, Clayton, Australia

**Keywords:** endometriosis, endometrial stem cells, SSEA1, SOX9, endometrial epithelium, regeneration, baboon model

## Abstract

**STUDY QUESTION:**

Is endometriosis associated with abnormally located endometrial *basalis*-like (SSEA1+/SOX9+) cells in the secretory phase *functionalis* and could they contribute to ectopic endometriotic lesion formation?

**SUMMARY ANSWER:**

Women with endometriosis had an abnormally higher number of *basalis*-like SSEA1+/SOX9+ epithelial cells present in the *stratum functionalis* and, since these cells formed 3D structures *in vitro* with phenotypic similarities to ectopic endometriotic lesions, they may generate ectopic lesions following retrograde menstruation.

**WHAT IS KNOWN ALREADY:**

Endometrial *basalis* cells with progenitor potential are postulated to play a role in the pathogenesis of endometriosis and SSEA1 and nuclear SOX9 (nSOX9) mark *basalis* epithelial cells that also have some adenogenic properties *in vitro*. Induction of ectopic endometriotic lesions in a baboon model of endometriosis produces characteristic changes in the eutopic endometrium. Retrograde menstruation of endometrial *basalis* cells is proposed to play a role in the pathogenesis of endometriosis.

**STUDY DESIGN, SIZE, DURATION:**

This prospective study included endometrial samples from 102 women with and without endometriosis undergoing gynaecological surgery and from six baboons before and after induction of endometriosis, with *in vitro* assays examining the differentiation potential of human *basalis*-like cells.

**PARTICIPANTS/MATERIALS, SETTING, METHODS:**

The study was conducted at a University Research Institute. SSEA1 and SOX9 expression levels were examined in human endometrial samples from women aged 18–55 years (by immunohistochemistry (IHC) and qPCR) and from baboons (IHC). The differential gene expression and differentiation potential was assessed in freshly isolated SSEA1+ endometrial epithelial cells from women with and without endometriosis (*n* = 8/group) *in vitro*. *In silico* analysis of selected published microarray datasets identified differential regulation of genes of interest for the mid-secretory phase endometrium of women with endometriosis relative to that of healthy women without endometriosis.

**MAIN RESULTS AND THE ROLE OF CHANCE:**

Women with endometriosis demonstrated higher number of *basalis*-like cells (SSEA1+, nSOX9+) in the *functionalis* layer of the eutopic endometrium compared with the healthy women without endometriosis in the secretory phase of the cycle (*P* < 0.05). Induction of endometriosis resulted in a similar increase in *basalis*-like epithelial cells in the eutopic baboon endometrium. The isolated SSEA1+ epithelial cells from the eutopic endometrium of women with endometriosis had higher expression of *OCT4, NANOG, FUT4* mRNA (*P* = 0.05, *P* = 0.007, *P* = 0.018, respectively) and they differentiated into ectopic endometriotic gland-like structures in 3D culture, but not into mesodermal lineages (adipose or bone cells).

**LARGE SCALE DATA:**

N/A

**LIMITATIONS, REASONS FOR CAUTION:**

Small sample size. Bioinformatics analysis and results depends on the quality of published microarray datasets and the stringency of patient selection criteria employed. Differentiation of SSEA-1+ cells was only examined for two mesodermal lineages (adipogenic and osteogenic).

**WIDER IMPLICATIONS OF THE FINDINGS:**

Since endometrial epithelial cells with SSEA1+/nSOX9+ *basalis*-like phenotype generate endometriotic gland-like structures *in vitro*, they may potentially be a therapeutic target for endometriosis. An in depth analysis of the function of *basalis*-like eutopic endometrial epithelial cells might provide insights into their potential deregulation in other disorders of the endometrium including heavy menstrual bleeding and endometrial cancer where their function may be aberrant.

**STUDY FUNDING/COMPETING INTEREST(S):**

We acknowledge the support by Wellbeing of Women project grant RG1073 (D.K.H., C.E.G.) and R01 HD083273 from the National Institutes of Health (A.T.F.). We also acknowledge the support of Liverpool Women’s Hospital Foundation Trust (J.D.), Institute of Translational Medicine (L.D.S., H.A.L., A.J.V., D.K.H.), University of Liverpool, the National Health and Medical Research Council of Australia ID 1042298 (C.E.G.) and the Victorian Government Operational Infrastructure Support Fund. All authors declare no conflict of interest.

## Introduction

Endometriosis is a common gynaecological disease, defined by the presence of benign endometrial epithelial and stromal cells outside of the uterine cavity. The ectopic (extra-uterine) endometriotic lesions retain features of eutopic (uterine) endometrial cells, such as oestrogen dependency and thus undergo cyclical growth and regeneration, resulting in inflammation and scar/adhesion formation. While the pathogenesis of endometriosis is not fully understood (Sourial *et al.*, 2014), the most commonly accepted theory is retrograde menstruation. This involves the trans-tubal migration and engraftment of viable endometrial fragments to the peritoneal mesothelium, establishing ectopic endometriotic lesions (Sampson, 1927). The theory is limited in that retrograde menstruation is a natural phenomenon, and has been demonstrated in over 90% of women (Blumenkrantz *et al.*, 1981), yet the prevalence of endometriosis in the general premenopausal female population is estimated only at 5–10% (Halme and Surrey, 1990). Such a discrepancy suggests that the aetiology is more complex. Adult stem/progenitor cells are believed to be responsible for the remarkable regenerative capacity of human endometrium, and are thus also implicated in proliferative endometrial pathologies, such as endometriosis (Gargett *et al.*, 2008; Valentijn *et al.*, 2013; Sourial *et al.*, 2014; Tempest *et al.*, 2018).

Epithelial cells with progenitor function are postulated to be located in the *basalis* layer of the premenopausal endometrium (Prianishnikov, 1978; Schwab *et al.*, 2008; Valentijn *et al.*, 2013; Tempest *et al.*, 2018). Leyendecker and colleagues further developed the theory of retrograde menstruation by proposing that women with endometriosis shed a higher number of *basalis*-like cells together with their eutopic endometrial *functionalis*, which then initiate ectopic endometriotic deposits (Leyendecker *et al.*, 2002). The group also hypothesised that secretory phase *functionalis* endometrium from women with endometriosis contain more ‘*basalis*-like cells’ and subsequently tried to characterise such cells on the basis of steroid receptor expression. However, since steroid receptors are expressed by cells located in both *basalis* and *functionalis* layers of the normal endometrium, the theory remains un-proven.

**Table I:** Demographic data.

	Endometriosis (*n* = 44)	Healthy Control (*n* = 58)	*P*-value (Mann–Whitney test)
Age (y)	38 (21–48)	41.5 (21–50)	*P* < 0.01
BMI (kg/m^2^)	25.5 (17.1–40.6)	26.8 (18.9–52.2)	n/s
Parity (%)			*P*
0	22/44 (50%)	4/58 (7%)	
1	9/44 (20%)	6/58 (10%)	
2	9/44 (20%)	23/58 (40%)	
>2	3/44 (7%)	24/58 (41%)	
Unknown	1/44 (2%)	1/58 (2%)	
Smokers	12/44 (27%)	15/58 (26%)	n/s
Endometriosis stage	2 (1–4)		n/a

Data expressed as median and range except smoking status and parity.

We have previously shown that cells with an SSEA1/nSOX9+ signature which are abundant in the *basalis* region of the eutopic endometrium, have some *in vitro* progenitor activity and a similar epithelial cell phenotype is observed in ectopic endometriotic lesions (Valentijn *et al.*, 2013). Furthermore, epithelial progenitor cell activity has been either demonstrated or proposed in endometrial epithelial cells expressing *N*-cadherin (Nguyen *et al.*, 2017), Musashi-1 (Gotte *et al.*, 2008; Tempest *et al.*, 2018), *LGR5* (Tempest *et al.*, 2018) and in a small proportion of side population (SP) cells (Masuda *et al.*, 2010; Cervello *et al.*, 2011). Ectopic endometriotic lesions also express Musashi-1 (Gotte *et al.*, 2008; Tempest *et al.*, 2018).

The baboon model of induced endometriosis simulates retrograde menstruation by the intra-peritoneal inoculation of menstrual endometrium curetted on menstrual Day 2 of the cycle, in two consecutive cycles. The model has complete success at inducing endometriosis both macro- and microscopically at 3 months (Harirchian *et al.*, 2012), and has thus facilitated understanding of the chronological changes in the disease process showing subsequent alterations in both the eutopic and ectopic endometrium after the establishment of initial ectopic endometriotic lesions (Braundmeier and Fazleabas, 2009; Hapangama *et al.*, 2010).

In this present study, we aimed to investigate the role that the previously characterised SSEA1+/nSOX9+ endometrial epithelial cells (Valentijn *et al.*, 2013) play in the pathogenesis of endometriosis by addressing the following research questions:

- Do women with endometriosis have an abnormally high number of SSEA1+/nSOX9+ *basalis*-like epithelial cells located in the secretory phase *functionalis* layer of the eutopic endometrium compared with healthy fertile women without endometriosis?
- Does induction of ectopic endometriotic lesions increase SSEA1+/nSOX9+ epithelial cells in the eutopic endometrium in a baboon model, simulating human disease?
- Are there differences in gene expression in SSEA1+ eutopic endometrial epithelial cells from women with and without endometriosis?
- What is the differentiation potential of purified SSEA1+ endometrial epithelial cells and can they produce gland-like structures similar to ectopic endometriotic lesions *in vitro*?

## Materials and Methods

### Human tissue collection

Collection of human endometrium was approved by Liverpool Adult Ethics committee (REC references; 09/H1005/55 and 11/H1005/4) and 89 human endometrial samples from pre-menopausal women (who had not been on hormonal treatments in the preceding 3 months) undergoing hysterectomy or laparoscopy were collected. From hysterectomy specimens, a wedge of tissue from the lumen to the muscular myometrial layer that included superficial and basal endometrium as well as myometrium was taken. A pipelle endometrial sampler was used to sample the endometrial *functionalis* layer of women undergoing laparoscopy. Further demographic information on patient groups is included in Table I and Supplementary Table SI.

### Induction of endometriosis in the baboons

The previously described technique of intra-pelvic autologous inoculation of curetted menstrual endometrium was employed to induce endometriosis in six female baboons with regular menstrual cycles (Fazleabas *et al.*, 2002). All tissues (ectopic and eutopic) were collected between Days 9–11 post-ovulation, which corresponds to the window of implantation (WOI) in the baboon, after determining the day of ovulation by serum E2 levels as previously described (Fazleabas *et al.*, 2002). This phase of the cycle where most known features of endometriosis associated changes in the eutopic endometrium is described was chosen for our study. Briefly, prior to the induction of the disease, eutopic endometrium was obtained from five of the six baboons. Following a 3 month rest period, laparoscopic autologous instillation of trans-cervically harvested menstrual endometrium using a Unimar Pipelle (suction curette) (Day 2) into the pouch of Douglas, the left and right cul-de-sac as well as the broad ligaments near the fallopian tubes of the same baboon on two consecutive menstrual cycles. Following the second inoculation, tissue was harvested from the eutopic endometrium and ectopic lesions after 3 months (*n* = 6) by performing laparoscopies and laparotomies as previously described (Fazleabas et al., 2002) and one lesion per animal per time point was analysed. Control endometrial tissues were also obtained at a single time point from eight additional normally cycling baboons that had not been inoculated with menstrual tissue. All animals weighed between 12 and 18 kg, were aged 7–12 years, and included tissue from previous studies (Fazleabas *et al.*, 2002) and tissues that were collected prospectively. The induction of the disease had no effect on their menstrual cyclicity or peripheral ovarian steroid levels (Wang *et al.*, 2009). The Animal Care Committees of the University of Illinois, Chicago and Michigan State University, approved all experimental procedures on baboons (Fazleabas *et al.*, 2002)

### Immunohistochemistry (IHC)

After collection, both human and baboon tissues were fixed in 10% (v/v) neutral buffered formalin for 24 h dehydrated through ethanol, embedded in paraffin and 3 μm sections prepared for IHC. Standard IHC was performed using heat induced antigen-retrieval and DAB chromogen as previously described (Hapangama *et al.*, 2010; Valentijn *et al.*, 2013). Immunostaining for all antibodies was analysed with specific reference to the two different epithelial compartments, the *functionalis* (typically in a secretory phase sample, glands in the upper 2/3 of the endometrium below the luminal epithelium, surrounded by sparse stroma) and the *basalis* (glands in the lower 1/3 of the endometrium adjacent to the endo-myometrial junction, surrounded by densely packed stroma) in full thickness hysterectomy endometrial tissue sections. The SSEA1 and SOX9 expressing epithelial cells were quantified using a modified Quickscore method which incorporates both staining intensity (0 = negative, 1 = weak, 2 = moderate, 3 = strong) and abundance (1 ≥ 0–25%, 2 ≥ 25–50%, 3 ≥ 50–75%, 4 ≥ 75–100%). The intensity and percentage scores were then multiplied and summed to give scores in the range 0–12 as previously described (Schiessl *et al.*, 2009; Valentijn *et al.*, 2013; Mathew *et al.*, 2016). Antibodies used for IHC are detailed in Supplementary Table SII.

### Cell sorting and analysis

Magnetic bead sorting (MACS) of single-cell epithelial suspensions from endometrial samples were labelled with anti-SSEA1 (CD15) MicroBeads (#130-094-530, Miltenyi Biotec, UK) and separated using MACS separation columns (MS columns, Miltenyi Biotec, UK) according to the manufacturer’s instructions and purity assessed as previously reported (Valentijn *et al.*, 2013).

### RNA extraction and cDNA synthesis

Total RNA from clinical tissue samples was extracted using TRIzol® Plus RNA Purification System (Life Technologies, Paisley, UK) according to the manufacturer’s instructions. The quantity of total RNA was determined by NanoDrop ND-1000 (ThermoFisher Scientific, UK). Total RNA was reverse transcribed using AMV First Strand cDNA synthesis kit (New England Bio Labs, Hertfordshire, UK). cDNA was amplified by PCR using HotStart Taq (New England Bio Labs, Hertfordshire, UK).

TRIzol® Reagent (Life Technologies) was used to isolate total RNA which was precipitated from samples according to the manufacturer’s instructions. Total RNA was extracted from whole tissue, SSEA1 sorted (MACS) cells and treated/untreated Ishikawa cells (positive control) and reverse-transcribed into cDNA using the Superscript III First-Strand Synthesis System (Invitrogen).

### Quantitative real time polymerase chain reaction (q-PCR) and RT-PCR

Semi-quantitative RT-PCR and qPCR were performed as previously described (Hapangama *et al.*, 2012; Valentijn *et al.*, 2013) using KAPA SYBR FAST qPCR Mix Master 2x (Kapa BioSystems) and the Rotor-Gene 3000 centrifugal real-time cycler (Corbett Research). Relative gene expression was calculated and normalised to the reference gene, YWHAZ due to its stability in the endometrium (Vestergaard *et al.*, 2011). The amplification products were verified using agarose gel electrophoresis, stained with SYBR Safe (Life Technologies, Paisley, UK) and visualised by UV transillumination using ChemiDoc-It TS2 Imager (UVP systems, Cambridge, UK). The PCR primers (Sigma-Aldrich) used are listed in Supplementary Table SIII.

### 3D epithelial cell cultures in Matrigel

Short-term cultured (16–36 h post-plating) MACS-bead sorted eutopic endometrial epithelial cells were trypsinised and re-suspended as single cell suspensions at ~100 000 cells/200 μl in undiluted Matrigel (BD Biosciences) and diluted with epithelial media serially two-fold to ~3000 cells/100 μl: 50 μl of the resulting mixture was plated in duplicate in 24-well tissue culture plates. After allowing the Matrigel to set at 37°C for 15–20 min, DMEM/F12 medium supplemented with insulin-transferrin-selenite (ITS, Invitrogen) and 50 ng/ml EGF (Sigma-Aldrich) was added. Medium was replaced every 3 days and cultures monitored over 14 days.

For IHC and immunofluorescence (IF), 3-D cultures were fixed in 10% (v/v) neutral-buffered formalin (NBF) for 30 min, harvested into 1% agarose in PBS and placed in NBF overnight at 4°C, then processed to paraffin wax, 3 μm sections were cut and antigen-retrieval performed (Supplementary Table SII). 3-D morphology and polarity was assessed with antibodies to MUC-1, laminin, cytokeratin 18 and β-catenin (Supplementary Table SII) (Valentijn *et al.*, 2013).

### Differentiation assay

#### Multi-lineage differentiation

Freshly harvested human eutopic endometrial epithelial cells were MACS-bead-sorted into SSEA1+ and SSEA1– fractions before placing in 2D culture in adipogenic and osteogenic media to assess their adipogenic or osteogenic differentiation potential as described below.

#### Adipogenic assay

Confluent cells were stimulated with *adipogenic media* every 2–3 days (High glucose DMEM/F12 (Lonza), 0.2% Primocin (Bioscience LIfesciences), 500 μM IBMX (Sigma-Aldrich), 1 μM Dexamethaxone (Sigma-Aldrich), 10 μM Insulin (Sigma Aldrich) (Gargett *et al.*, 2009). Non-stimulated cells were cultured in the same *epithelial media* as in the 3D culture and served as a negative control. After 2 weeks, cells were washed twice with PBS and fixed by incubation with 4% para-formaldehyde (PFA; Sigma-Aldrich) for 10 min, for analysis. Oil Red O staining was used to confirm the presence of lipid droplets. Briefly, PBS was removed from fixed cells and replaced with 60% isopropanol (Sigma-Aldrich). After 10 min, 60% Oil Red O stain solution was added and left for another 10 min until washed with water. Cells were counterstained with Gills 2 haematoxylin (Thermo Scientific). Images were visualised and captured with the use of a Nikon Biophot Microscope and camera head (Nikon).

#### Osteogenic assay

Near confluent cells were stimulated every 2–3 days with *osteogenic medium* for 2 weeks (High glucose DMEM/F12 (Lonza), 0.01 μM Vitamin D3 (Sigma-Aldrich), 50μM l-ascorbic acid (Sigma-Aldrich), 10 mM β-glycerol phosphate (Sigma-Aldrich) (Gargett *et al.*, 2009)). Cells stimulated with *epithelial medium* for the same period served as a negative control. On Day 15, cells were fixed by incubation with 4% PFA at room temperature for 10 min and subjected to Alkaline phosphatase (ALP) staining. Accordingly, PBS was aspirated from fixed cells and replaced with 400 μl of Fast Red/Napthol solution (Sigma-Aldrich) and left to incubate at room temperature. After 30 min, cells were rinsed with 0.1MTris HCl (pH 9.2) and PBS before counterstaining with 4′,6-diamidino-2-phenylindole (DAPI).

### hMSC cell culture

Human mesenchymal stem cells (hMSCs) were used as the positive control for *in vitro* differentiation into adipocytes and osteocytes (Lonza, Walkersville, Inc., USA). hMSC were cultured in Growth MediumTM (hMSCGM; Lonza, Walkersville Inc., USA) and maintained at 37°C, at 5% CO_2_ in air.

### Systems biology

The differential expression of epithelial specific *SOX9* and all fucosyltransferase (*FUT)* genes including *FUT3 and FUT4*, the enzymes which catalyse the addition of the fucosyl moiety to the Lewis X molecule that comprises the SSEA-1 epitope, as well as the differential expression of a total of 595 genes regulated by SOX9 in two gene sets (set 1 = 237 genes, set 2 = 358 genes from Illumina’s BaseSpace Correlation Engine) in the secretory endometrium was examined in three published whole transcriptomic RNA Expression studies examining the human mid-secretory endometrium of women with endometriosis (1) moderate-severe (*n* = 9, GSE6364 (Burney *et al.*, 2007)), (2) mild (*n* = 9, GSE51981 (Tamaresis *et al.*, 2014)), (3) moderate (*n* = 18, GSE51981 (Tamaresis *et al.*, 2014)) endometriosis suffers against healthy women without endometriosis (GSE6364, *n* = 8; GSE51981, *n* = 8) using Illumina’s BaseSpace Correlation Engine (BSCE; (Kupershmidt *et al.*, 2010) software;https://www.illumina.com/informatics/research/biological-data-interpretation/nextbio.html, last accessed on August 8, 2018; Illumina, San Diego, CA, USA). We also interrogated gene lists (already published) in Supplementary Table SII of Afshar *et al.*, 2013) from microarray experiments comparing secretory phase baboon eutopic endometrium from animals having either spontaneous or induced endometriosis with healthy animals.

### Statistical analysis

All statistical analyses were performed using GraphPad Prism software. Summary statistics and paired *t*-test or non-parametric equivalent (Mann–Whitney *U* and Spearman Rank) was employed as appropriate. Data are presented as median and interquartile range as indicated. Results were considered statistically significant when *P* < 0.05.

## Results

### Women with endometriosis show an increased number of SSEA1 and nSOX9 expressing basalis-like cells located in the functionalis layer of secretory phase eutopic endometrium

**Figure 1:**
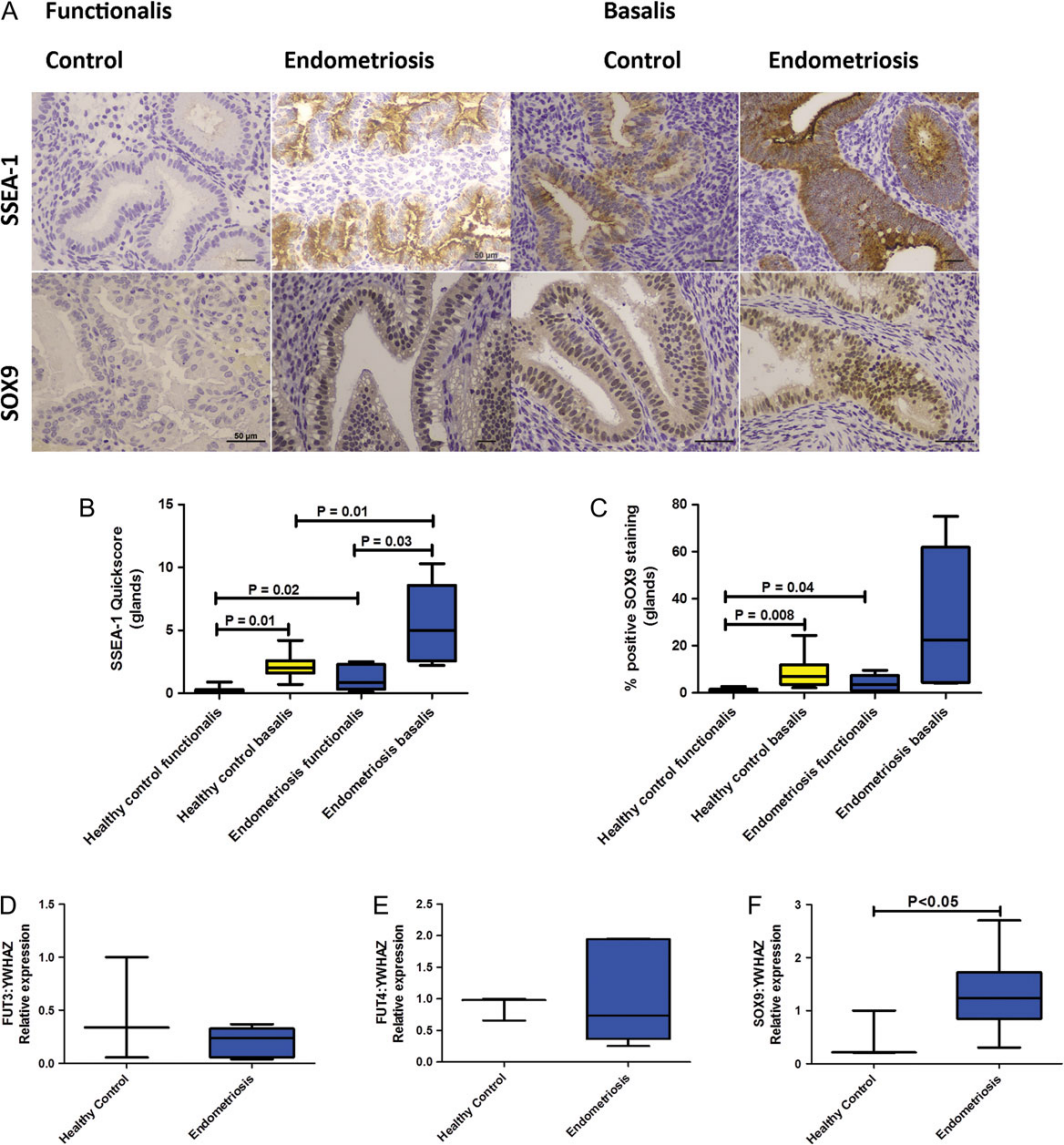
**Immunohistochemical staining for endometrial SSEA-1 and SOX9 in normal fertile women without endometriosis (*n* = 14) and women with surgically confirmed symptomatic endometriosis (*n* = 10).** Representative 400× micrographs showing the *basalis* and *functionalis* staining (**A**). Scale bar is 50 μm. Graphs showing immunohistochemical staining quickscores for SSEA1 (**B**) and SOX9 (**C**) protein expression in endometrial *functionalis* of healthy control women and endometriosis patients in the secretory phase of the cycle. Charts display median and quartiles with whiskers showing the range. Graphs depicting the enzymes likely responsible for catalysing the SSEA-1 epitope, *FUT3* (**D**), *FUT4* (**E**), and *SOX9* ( **F**) mRNA expression in healthy fertile control tissue (*n* = 3) and endometriosis patient samples (*n* = 6). Charts display median and quartiles with whiskers showing the range but just median and range when *n* = 3.

We tested the hypothesis that women with endometriosis have an increased number of *basalis*-like cells that will be shed in the subsequent menses. Significantly increased epithelial quick-scores for SSEA1 and nSOX9 were observed in the *functionalis* layer of secretory phase eutopic endometrium in women with endometriosis compared with healthy fertile control women; (SSEA1 median = 0.85, IQR = 0.33–2.28, versus median=0.01 IQR = 0–0.28, *P* = 0.02, (Fig. 1A and B)) and nSOX9 (median=3.6, IQR = 1–7.4 versus median=0.60, IQR = 0.19–1.58, *P* = 0.04) (Fig. 1C).

The women with endometriosis also had higher *basalis* epithelial quickscores in the secretory endometrium when compared with the healthy fertile control women, for SSEA1 (median=5.00, IQR = 2.6–8.6 versus median=2.00, IQR = 1.6–2.6; *P* = 0.01) (Fig. 1B). Although a similar trend was observed with nSOX9 it was not statistically significant (median=22.5, IQR = 4.5–61.8 versus median=7.1, IQR = 3.6–12; *P* = 0.3) (Fig. 1C).

To further understand the increased SSEA-1 immunostaining in endometriosis tissues, we assessed several fucosyl transferase enzymes (*FUT3, FUT4*) to determine if these differed. The FUTs enzymatically add the SSEA-1 epitope to Lewis X molecule. The transcript for *FUT3* and *FUT4* used as a surrogate for SSEA1 epitope did not change in the endometrial tissue derived from women with endometriosis (*n* = 6) compared with the healthy fertile control samples (*n* = 3) (Fig 1D and E). In contrast, *SOX9* mRNA levels were significantly higher in tissue from women with endometriosis (median=1.2, IQR = 0.85–1.7, *n* = 6) compared with the healthy fertile control samples (median 0.2, IQR = 0.2–1, *n* = 3, *P* < 0.05, Fig. 1F). *In silico* interrogation of published curated datasets also revealed that several FUT genes that could be responsible for the SSEA1 epitope (including *FUT3, FUT4* as well as *FUT2, FUT5, FUT6, FUT7)* and *SOX9* gene were up-regulated (and 402/595 of SOX9 regulated genes were differentially regulated) in mid-secretory phase endometria of women with endometriosis compared with the samples of healthy women without endometriosis (Supplementary Figure S1).

Both SOX9 and SSEA1 antigen expression were high in human ectopic lesions (Supplementary Figure S2B, as previously shown in Valentijn *et al.*, 2013) suggesting a possible functional role for these in the ectopic lesions.

### Eutopic endometrial SSEA1 and SOX9 expression changed with the induction of endometriosis in a baboon model

**Figure 2:**
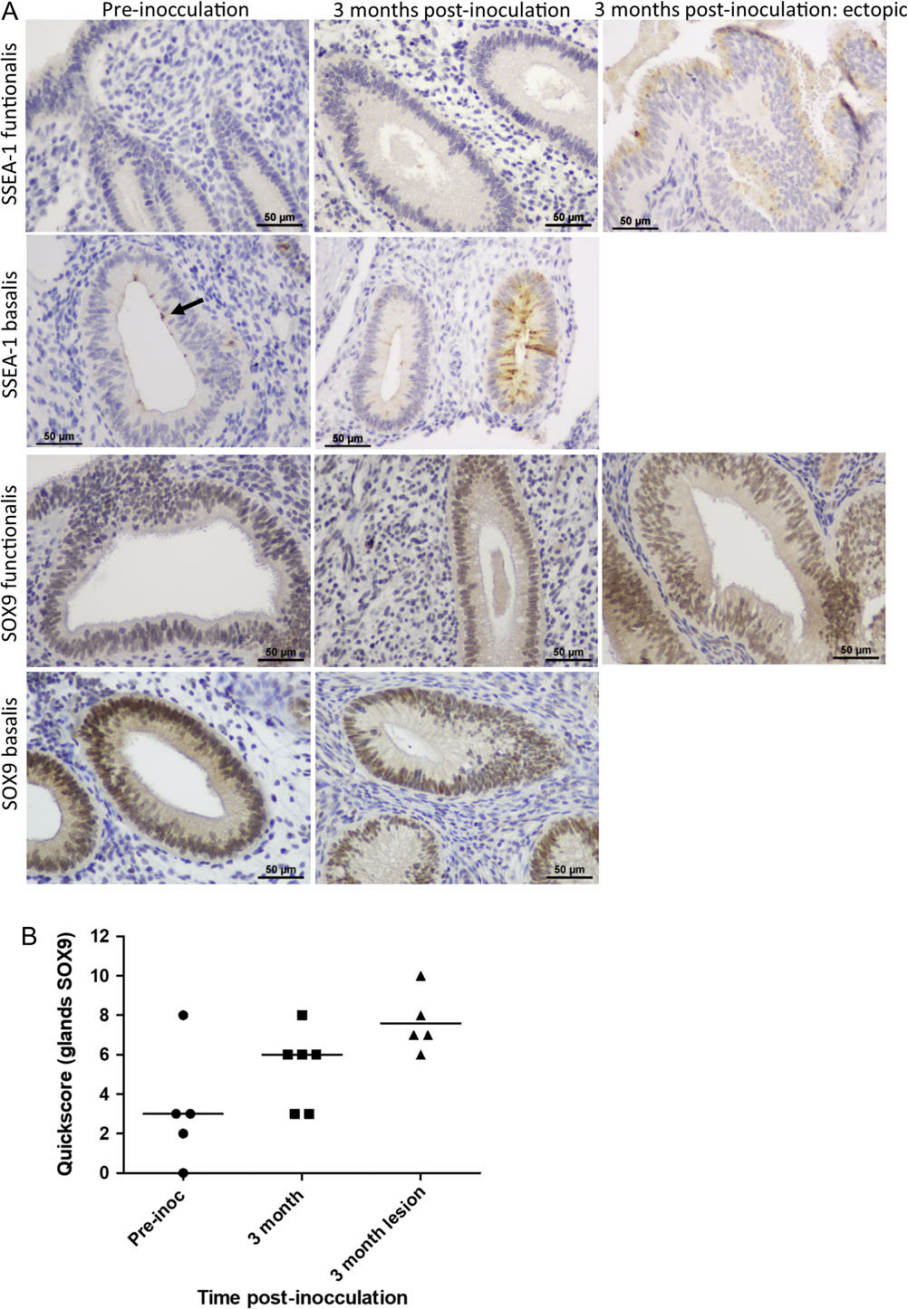
**Representative micrographs showing SSEA1 and SOX9 in the baboon model of endometriosis** (**A**). Scale bar is 50 μm. Graph shows *functionalis* glandular SOX9 quickscore and median values (**B**) for control (pre-inoculation) baboons (*n* = 5) and 3 months post-inoculation (both eutopic endometrium (*n* = 6) and ectopic lesions (*n* = 5),). Mann–Whitney *U* test showed no statistically significant difference in SOX9 quickscore between control and 3 months post-inoculation for eutopic endometrium (*P* = 0.2) or ectopic lesions (*P* = 0.07) respectively.

We hypothesised that the baboon model would allow examination of chronological changes in basalis-like cells in ectopic and eutopic endometrium after induction of endometriosis. Most epithelial cells of the baboon ectopic endometriotic lesions had high nSOX9 immunostaining confirming similarities with the previously published human data (Valentijn *et al.*, 2013) (Fig. 2A). However, only two of four baboon ectopic endometriotic lesions examined showed high SSEA1 expression (Fig. 2A, *P* > 0.05).

Three months after endometriosis induction, eutopic endometrial *functionalis* also showed apparently higher but not significant quick-scores for nSOX9, at or above the median pre-inoculation scores in all animals (Fig. 2B, *P* > 0.05). The control baboons showed similar endometrial SOX9 expression pattern to the healthy humans with high nSOX9 expression observed in the *basalis* glands (Fig. 2A).

Three months after induction of endometriosis, SSEA1 expression increased in the eutopic endometrial *basalis* in 4/6 (67%) of the animals but unlike normal human *basalis* glands, only a very few discreet SSEA1 expressing cells were observed in the eutopic endometrial *basalis* of the control baboons (Fig. 2A). Specifically, by comparing the previously published microarray data from eutopic endometrium from baboons after induction of endometriosis with the pre-inoculation endometrium of the same animals, we demonstrated increased expression of *SOX9* and many of the FUT genes (including *FUT3, FUT5, FUT8* and *FUT11* that could be responsible for SSEA1 epitope expression) in baboons.

### Basalis-like (SSEA1+) epithelial cells of women with endometriosis showed differential gene expression compared with the healthy women

**Figure 3:**
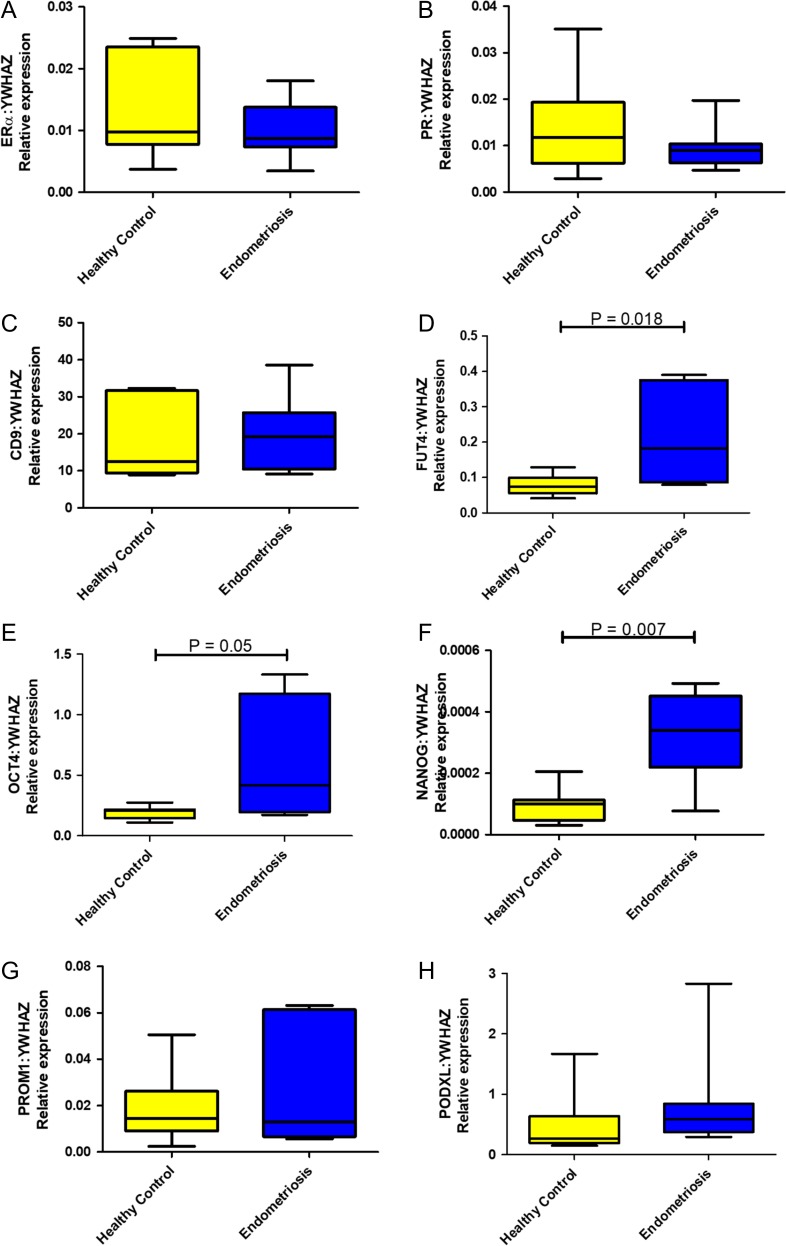
**Quantitative RT-PCR results from SSEA1+ sorted epithelial cells from women with endometriosis (*n* = 8) and control women (*n* = 12) showing relative expression of ERα (*ESR1*, **A**), *PGR* (**B**), *CD9* (**C**), *FUT4* (**D**, Mann–Whitney *U* test *P* = 0.018), *OCT4* (**E**, Mann–Whitney *U* test *P* = 0.05), *NANOG* (**F**, Mann–Whitney* U* test *P* = 0.007)*, PROM1* (**G**) and *PODXL* (**H**).** All data is shown relative to the housekeeping gene *YWHAZ*.

Since endometriosis may result in retrograde flow of the *basalis*-like cells at menses, we next attempted to enrich for SSEA1+ *basalis* epithelial cells derived from the eutopic endometrium of women with and without endometriosis to assess their expression of a panel of selected genes. Comparison of SSEA1+ sorted epithelial cells from women with endometriosis and healthy fertile control women (*n* = 8/group), showed a significant up-regulation of two pluripotency genes *NANOG* (*P* = 0.007) and *OCT4 (P* = 0.05), (Fig. 3D,E) although the abundance of OCT4 was extremely low. The third gene required for pluripotency, *SOX2* was not expressed in whole healthy human endometrial tissue or in any of the endometrial epithelial fractions (data not shown) despite high expression in the positive control human embryonic stem cells. Immunohistochemistry staining for anti-SOX2 in full thickness endometrial samples revealed negative staining (Supplementary Fig. S3), indicating that SSEA1+ cells are unlikely to be pluripotent and neither are pluripotent stem cells found in human endometrium. mRNA for *FUT4*, was upregulated in SSEA1+ cells from women with endometriosis compared with those from normal endometrium (Fig 3D). There were no significant differences in the endometrial differentiation genes, *PR* or *ESR1* between endometriosis and normal SSEA1+ sorted cells (Fig. 3A,B). Neither was there any difference for the other epithelial genes that have been proposed as progenitor markers in other tissues assessed in our panel (*PROM1*, *CD9* and *PDXL*, Fig. 3D,G,H).

### SSEA1+ cells derived from the eutopic endometrium of women with endometriosis produce ectopic endometriotic lesion-like structures in 3D culture

**Figure 4:**
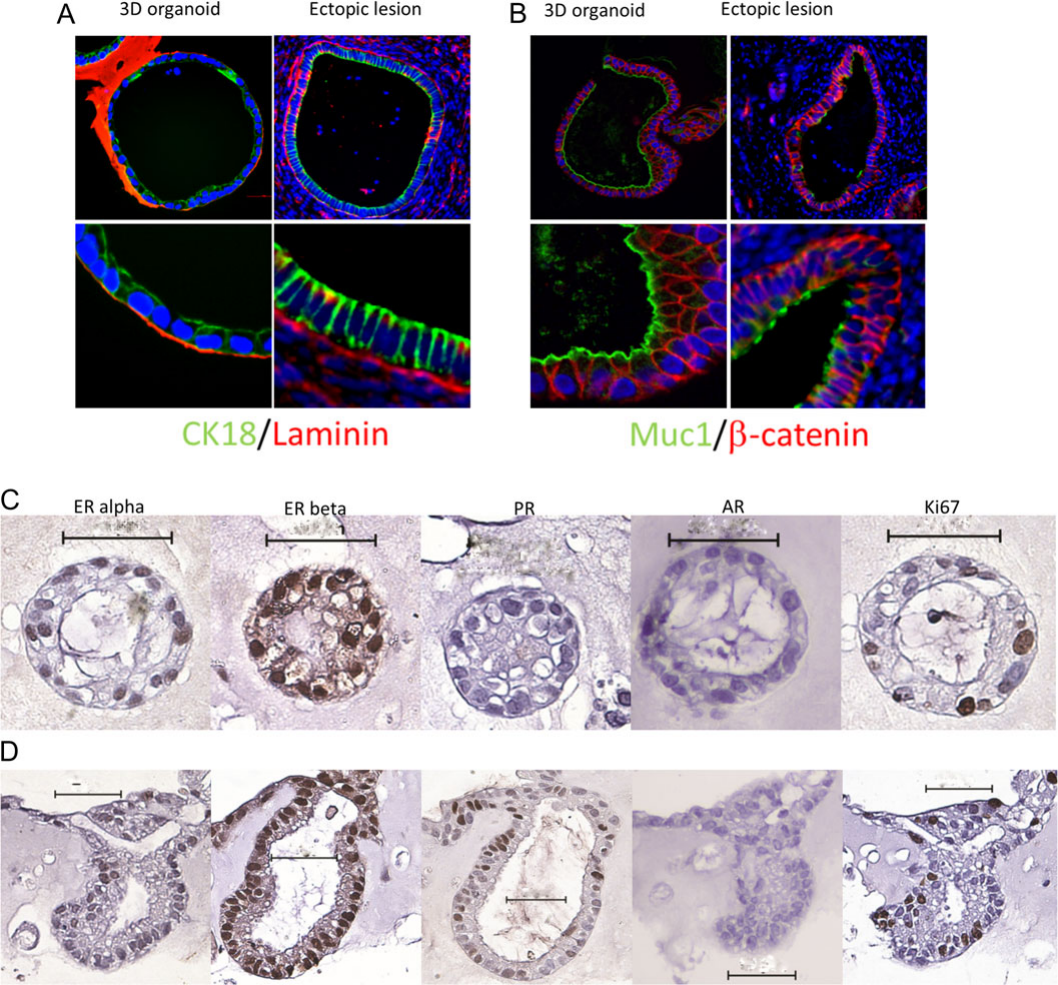
**Formalin-fixed and paraffin-embedded sections of gland-like structures grown in 3D culture and ectopic lesions stained by immunofluoresence, for cytokeratin18/laminin (**A**), MUC-1/β-catenin (**B**).** and for steroid receptors with immunohistochemistry show ERβ was the dominant receptor in both early immature non-polarised (**C**) and mature, polarised (**D**) gland-like structures. Whilst both these structures contained some cells expressing ERα and the proliferative marker Ki67, PR was only seen in the mature polarised structures. AR was not expressed by any epithelial cells grown in 3D. Scale bar = 50 μm.

We then tested the hypothesis that SSEA1+ cells are able to generate structures similar to ectopic endometriotic lesions *in vitro*. All sorted samples of SSEA1+ cells from eutopic endometrial cell suspensions of women with endometriosis generated gland-like structures within 14 days of 3D culture. These 3D structures morphologically mimicked ectopic endometrial epithelium (Fig. 4A and B). Co-localisation of the urogenital epithelial marker cytokeratin-18 with the adherins junction molecule β-catenin in ectopic lesions established their endometrial origin (Fig. 4A and B) and epithelial phenotype of the ectopic cells. MUC1 at the apical surface of the epithelia and basal laminin confirmed the polarisation and differentiation of the epithelial cells as well as their striking similarities with ectopic lesions (Fig. 4A and B). Immature gland-like structures (Fig. 4C) consisted of cells expressing mainly ERβ with occasional cells expressing ERα and the proliferative marker Ki67. However, the more mature larger gland-like structures demonstrated nuclear staining in some cells for progesterone receptor but AR was not expressed by the epithelial cells in 3D culture at any stage (Fig. 4C and D).

### SSEA1+ endometrial epithelial cells are distinct from mesenchymal cells and do not differentiate into mesodermal lineages *in vitro*

**Figure 5:**
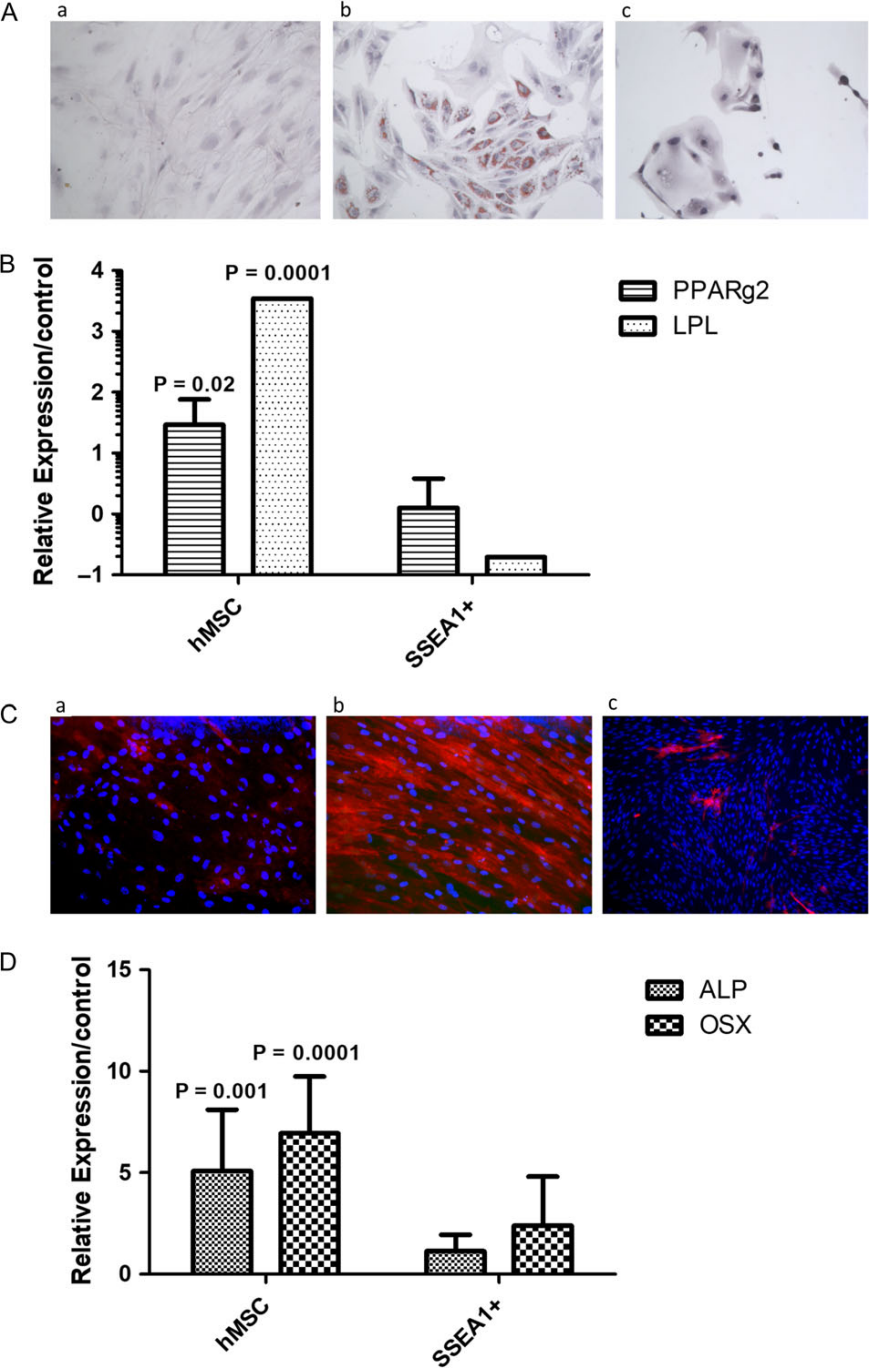
**Micrographs of staining and mRNA expression data depicting that SSEA1+ endometrial epithelial cells do not express markers of mesodermal differentiation when cultured in adipogenic and osteogenic media.** (**A**) Micrographs showing Oil O red staining for d14/15 hMSC in control medium (a), hMSC in adipogenic medium (b) SSEA1+ epithelial cells in adipogenic medium (c). Relative expression compared to control values were significantly higher in hMSC for *PPARg2* and *LPL* and unchanged in SSEA1+ sorted cells (**B**). (**C**) shows micrographs for alkaline phosphatase staining in d14/15 hMSC in control medium (a), hMSC in osteogenic medium (b) SSEA1+ epithelial cells in osteogenic medium (c) relative expression compared to control values were significantly higher in hMSC for the osteogenic markers alkaline phosphatase (*ALP*) and osterix (*OSX*), remaining unchanged in SSEA1+ sorted cells (**D**)

We then sought to confirm that the SSEA1+ *basalis*-like cells are not pluripotent and are likely to be unipotent, therefore if they reach the peritoneal cavity will probably produce an endometrial epithelial phenotype without differentiating into other mesenchymal cell types. To this end, we finally assessed whether MACS sorted-SSEA1+ *basalis-like* epithelial cells derived from the eutopic endometrium of women with endometriosis could differentiate into two representative mesodermal lineages. Human bone marrow-derived mesenchymal stem/stromal (hMSCs), which differentiate into adipocytes and osteoblasts served as a positive control. After 2 weeks in culture in appropriate induction media, hMSCs differentiated into adipocytes and osteocytes (Fig. 5Ab and Cb). In contrast, SSEA1+ cells showed no change in morphology and did not develop intracytoplasmic lipid droplets after two weeks of culture in adipogenic medium (Fig. 5Ac), nor did they show alkaline phosphatase activity after incubation in osteogenic medium (Fig. 5Cc). This was confirmed by negative staining with Oil Red-O and minimal expression, of early adipogenic markers PPARγ2 and LPL (Fig. 5B). Similarly, mRNA expression of early osteocyte differentiation markers (alkaline phosphatase, osterix) did not change in SSEA1+ endometrial epithelial cells (Fig. 5D).

## Discussion

**Figure 6:**
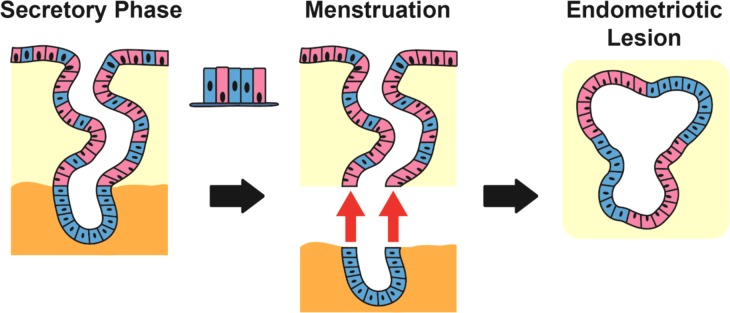
**Schematic illustration of the possible involvement of SSEA1+SOX9+ *basalis*-like cells (blue) in endometriotic ectopic lesion formation.** In women with endometriosis, *functionalis* layer containing increased *basalis*-like SSEA1+nSOX9+ (blue) cells will be shed at menstruation. Retrograde flow of these into the peritoneal cavity likely give rise to ectopic lesions and we have demonstrated the ectopic lesions to contain SSEA1+nSOX9+ epithelial cells.

This study examines the involvement of SSEA1+SOX9+ *basalis*-like epithelial cells derived from eutopic endometrium in the pathogenesis of endometriosis. We have shown that the eutopic endometrial *functionalis* epithelium of women with endometriosis, aberrantly contain an increased number of cells with SSEA1+SOX9+ *basalis*-like epithelial cell phenotype. The chronological changes occurring in the eutopic endometrium with induction of ectopic endometriotic lesions were examined in the baboon, where induction of endometriosis resulted in a trend to increased SOX9+ cells in eutopic endometrium. As previously described in human lesions, the baboon endometriotic lesions induced in ectopic sites also contained SSEA1+SOX9+ cells. SSEA1 enriched cells derived from the eutopic endometrium of women with endometriosis showed an increased level of expression of some ‘primitive’ genes compared with healthy women without endometriosis. These cells also produced 3D structures *in vitro* with phenotypic similarities to ectopic endometriosis lesions collected from women, suggesting that if they were deposited in the peritoneal environment (ectopic locations) after retrograde menstruation, they may initiate endometriotic lesions. SSEA-1+ cells derived from eutopic endometrium were unable to differentiate into the mesodermal lineages (bone and adipose), *in vitro* (Fig. 6).

Endometriosis is a disease associated with changes to eutopic endometrium that are most prominent in the progesterone dominant secretory phase of the cycle (Bulun *et al.*, 2006). The baboon model of endometriosis induction has demonstrated that establishment of endometriosis in ectopic sites induces aberrant expression of many genes and proteins that are characteristic of the eutopic endometrium in women with endometriosis and may contribute to functional consequences such as subfertility (Hastings and Fazleabas, 2006; Hapangama *et al.*, 2010; Sourial *et al.*, 2014; Afshar *et al.*, 2013). Interestingly, *SOX9* and some *FUTs* as well as *MSI1* (Musashi-1) were amongst these differentially expressed genes in baboons and these changes may be relevant to aberrations in the number and location of *basalis*-like epithelial cells and/or the increase in the progenitor cell activation/migration.

These changes in the eutopic endometrium may also promote the propagation of the disease (Hapangama *et al.*, 2010; Tempest *et al.*, 2018). Leyendecker *et al.*, proposed that women with endometriosis shed endometrial tissue with a *basalis*-like phenotype at menstruation (Leyendecker *et al.*, 2002), and therefore these cells are more likely to initiate ectopic lesions after retrograde menstruation. The recent discovery that SOX9 and SSEA1 are preferentially expressed in *basalis* epithelial cells (Valentijn *et al.*, 2013) provided us with a means to test Leyendecker’s theory and we have confirmed that women with endometriosis have an increased number of *basalis*-like cells in the secretory *functionalis* layer that will subsequently be shed at menstruation. The finding of numerous SSEA1+ cells in the *functionalis* of eutopic endometrium of women with endometriosis also suggests their delayed differentiation into SSEA1- *functionalis* epithelial cells given the recent descriptions of a potential hierarchy of epithelial cells from the basalis through the functionalis (Valentijn *et al.*, 2013; Nguyen *et al.*, 2017; Tempest *et al.*, 2018; Tempest *et al.*, 2018). This finding also agrees with the observation that the number of *basalis*-like cells increased in the endometrium of the baboons after inducing endometriosis. Interestingly, in the baboon model, an endometrial sampler was used to collect the exposed progenitor rich endometrial *basalis* on the second day of menstrual bleeding, and the subsequent placement of this tissue in the pelvic cavity resulted in 100% induction of endometriosis (Braundmeier and Fazleabas, 2009). This well-established method of endometriosis induction in the baboons confirms the involvement of *basalis*-like cells in the initial and subsequent ectopic endometriotic lesion formation and Leyendecker’s theory that *basalis*-like cells give rise to ectopic lesions. Furthermore, the previously published microarray data demonstrate, aberrant expression of these genes was persistently observed during disease progression after induction of endometriosis in baboons (already published in Supplementary Table SII of Afshar *et al.*, 2013).

FUT enzymes catalyse the addition of fucose to precursor polysaccharides in the last step of Lewis antigen biosynthesis, to generate fucosylated carbohydrate structures such as SSEA1. SSEA1 is reported to play a role in cell adhesion and regulation of cell differentiation (Eggens *et al.*, 1989, Kojima *et al.*, 1994). Interrogation of the microarray data provides validation of our data in independent external datasets, demonstrating differential expression of several FUTs in the human endometrium. Of particular interest, was the observation that in women with endometriosis, SSEA1+ epithelial cells had significantly elevated levels of *FUT4* compared with SSEA1+ epithelial cells from healthy women without endometriosis. Cells expressing the Lewis-x antigen (such as SSEA-1) when transfected with *FUT4* adopted a more adhesive phenotype in culture (Sudou *et al.*, 1995). Thus, increased *FUT4* expression in SSEA1+ cells in endometrial epithelial cells from endometriosis patients could similarly enhance their adhesive nature at ectopic sites. This effect of *FUT4* remains to be formally tested in endometrial epithelial cells, but we anticipate gain-of-function (overexpression) and loss-of function (small interfering RNA) studies to answer this in the future.

The transcription factors *NANOG, OCT4, SOX2* work synergistically at maintaining the pluripotent, embryonic stem cell phenotype (Kashyap *et al.*, 2009). The forced expression of these factors in somatic cells confers induced pluripotent stem cells status, exemplifying their importance, and therefore they are key markers of an undifferentiated state. The lack of concomitant *SOX2* expression with *NANOG* and *OCT4* in the healthy endometrial samples and isolated cells suggest that the adult healthy human endometrium may not contain pluripotent cells. We observed a higher *NANOG* and *OCT4* mRNA levels in the SSEA1+ cell fraction isolated from women with endometriosis, possibly suggesting that they are at an earlier stage of differentiation than those from women without endometriosis. It is tempting to postulate that such differences may further contribute to the ability of those cells in establishing an active endometriotic lesion after retrograde menstruation. This is corroborated by our subsequent experiment where all SSEA1+ sorted cell samples from women with endometriosis (*n* = 8) produced gland-like structures in 3D culture compared to the routine success of <70% in 3D cultures of singly dispersed SSEA1+ cells from healthy women without endometriosis (*n* > 20). However, we did not test this hypothesis formally therefore further studies are needed to confirm this observation.

The ectopic lesion-like structures grown in 3D, contained cells expressing all steroid receptors except AR. ERβ in particular, a receptor that is expressed by epithelial cells throughout the cycle as well as in hypo-oestrogenic postmenopausal endometrium, was highly expressed by the cells forming these gland-like structures (Hapangama *et al.*, 2015; Kamal *et al.*, 2016). ERβ is a major player in ectopic endometriotic lesion growth in many human and animal models (reviewed in Hapangama *et al.*, 2015; Simmen and Kelley, 2016) in agreement with our data.

The ability of singly dispersed SSEA1+ cells derived from eutopic endometrium to develop gland-like structures expressing a similar panel of markers to endometriotic lesions excised from women suggest a capability to generate these lesions in an ectopic environment *in vivo*. SSEA1+ cells were unable to differentiate into the two mesodermal lineages tested *in vitro* while assay success was confirmed in the control hMSCs. In contrast, endometrial stromal progenitors differentiate into adiopogenic, myogenic and osteogenic lineages (Schwab and Gargett, 2007). Previous heterotopic tissue reconstitution studies have also shown that adult endometrial epithelial cells are able to maintain the endometrial phenotype and morphogenesis, despite the origin of the mesenchymal stroma supporting the growth (Kurita *et al.*, 2001) suggesting that ectopic lesions retain the endometrial phenotype due to the unipotent epithelial progenitor cells that likely initiate them. Interestingly, a recent study analysing the presence of selection-neutral passenger mutations also suggested that epithelium in the endometriotic lesions was clonal and epithelial development is independent of the stroma (Noe *et al.*, 2018). It is however expected that the endometrial perivascular mesenchymal stem cells would facilitate the development of a vascular stroma also supporting the growth of endometriosis lesions (Cousins *et al.*, 2018; Tempest *et al.*, 2018).

Our data is of importance for many reasons; they provide further evidence for the involvement of *basalis*-like endometrial cells in the pathogenesis of endometriosis (Fig. 6); and they add to the existing data on endometriosis-associated eutopic endometrial aberrations. Our study, for the first time, provides an explanation of why 6–10% of women develop endometriosis when almost all undergo retrograde menstruation from a stem/progenitor cell perspective. Our data collectively suggest that shedding and retrograde menstruation of *basalis*-like cells is a prerequisite for ectopic lesion formation. In the context of endometrial epithelial progenitor/stem cells, it will be important in future studies to determine if the other suggested epithelial progenitor cells expressing *N*-cadherin (Nguyen *et al.*, 2017), or *LGR5* (Tempest *et al.*, 2018) play a role in the pathogenesis of endometriosis. Such studies will increase our current knowledge on the role of endometrial *basalis* epithelial stem/progenitor cells in endometrial proliferative disorders with potential to identify abnormalities in these *basalis* cell types and exploit them for diagnostic and therapeutic strategies in endometriosis as well as in other persistent endometrial proliferative disorders with a stem/progenitor cell basis.

## Supplementary Material

Supplementary Figure 1

Supplementary Figure 2

Supplementary Figure 3

Supplementary Table 1

Supplementary Table 2
