# Supplementary material for: Abnormally located SSEA1+/SOX9+ endometrial epithelial cells with a basalis-like phenotype in the eutopic functionalis layer may play a role in the pathogenesis of endometriosis
Source: Hum Reprod. 2018 Nov 29;34(1):56–68. doi: 10.1093/humrep/dey336 (PMC6295963; doi:10.1093/humrep/dey336)
Supplement: Supplementary Figure 1 [file dey336supplement_figure1.pdf]

Human Reproduction, 2018
doi:10.1093/humrep/dey336

human reproduction

SUPPLEMENTARY DATA

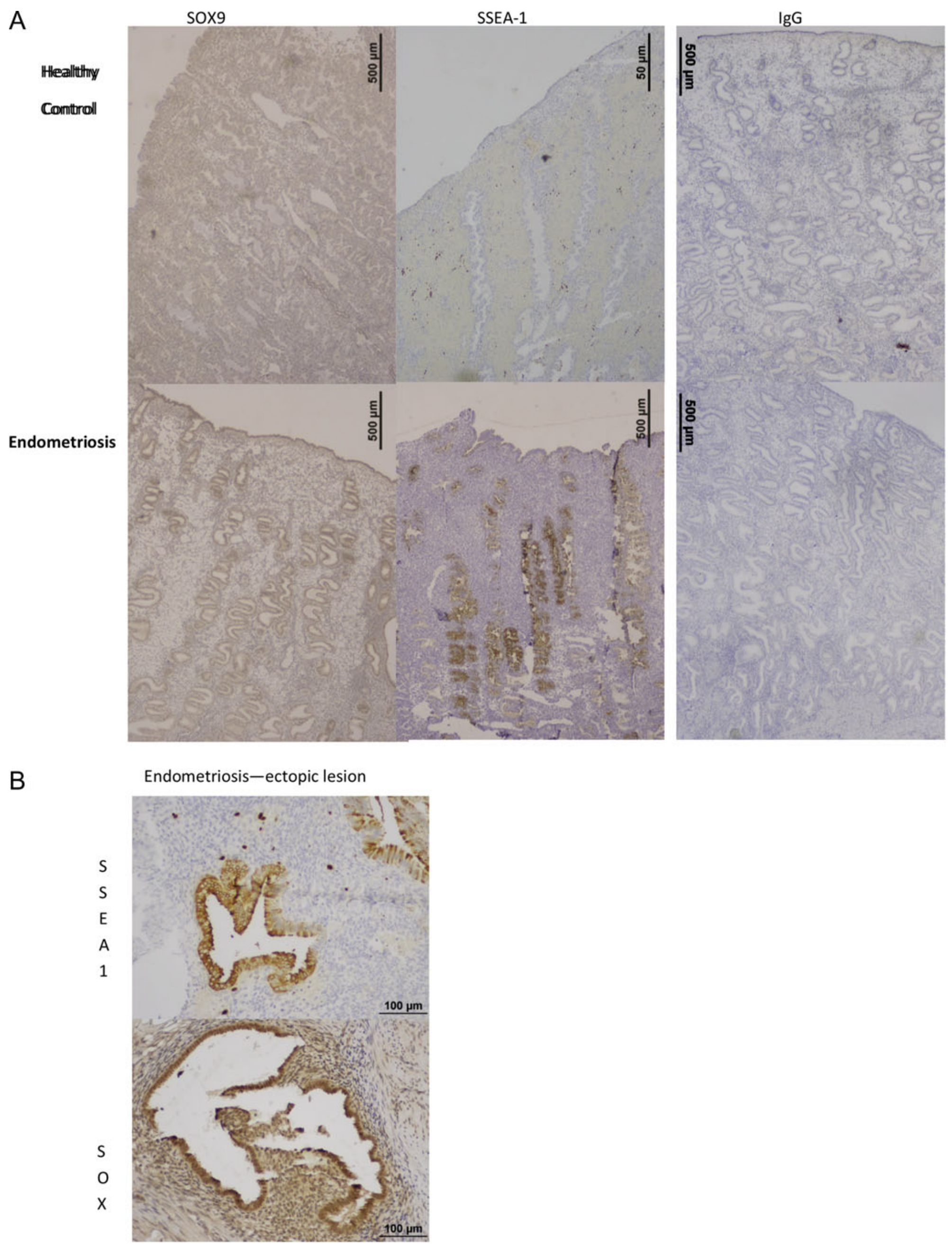

Supplementary Figure S1 Full-thickness endometrial samples from women with and without endometriosis in the secretory phase of the cycle stained for SSEA1, SOX9 and negative control IgG by immunohistochemistry. Scale bar = 500 µM. Upper IgG panel shows representative goat IgG negative control staining, with representative mouse IgG negative control staining below. Higher power micrographs are presented in Fig. 1A. Representative micrographs from ectopic endometriotic lesions stained by IHC for SSEA1 and SOX9 (Supplementary S1B). Seventeen ectopic lesions were examined from 13 women. Scale bar = 100 µm.
