# Supplementary material for: Abnormally located SSEA1+/SOX9+ endometrial epithelial cells with a basalis-like phenotype in the eutopic functionalis layer may play a role in the pathogenesis of endometriosis
Source: Hum Reprod. 2018 Nov 29;34(1):56–68. doi: 10.1093/humrep/dey336 (PMC6295963; doi:10.1093/humrep/dey336)
Supplement: Supplementary Figure 2 [file dey336supplement_figure2.pdf]

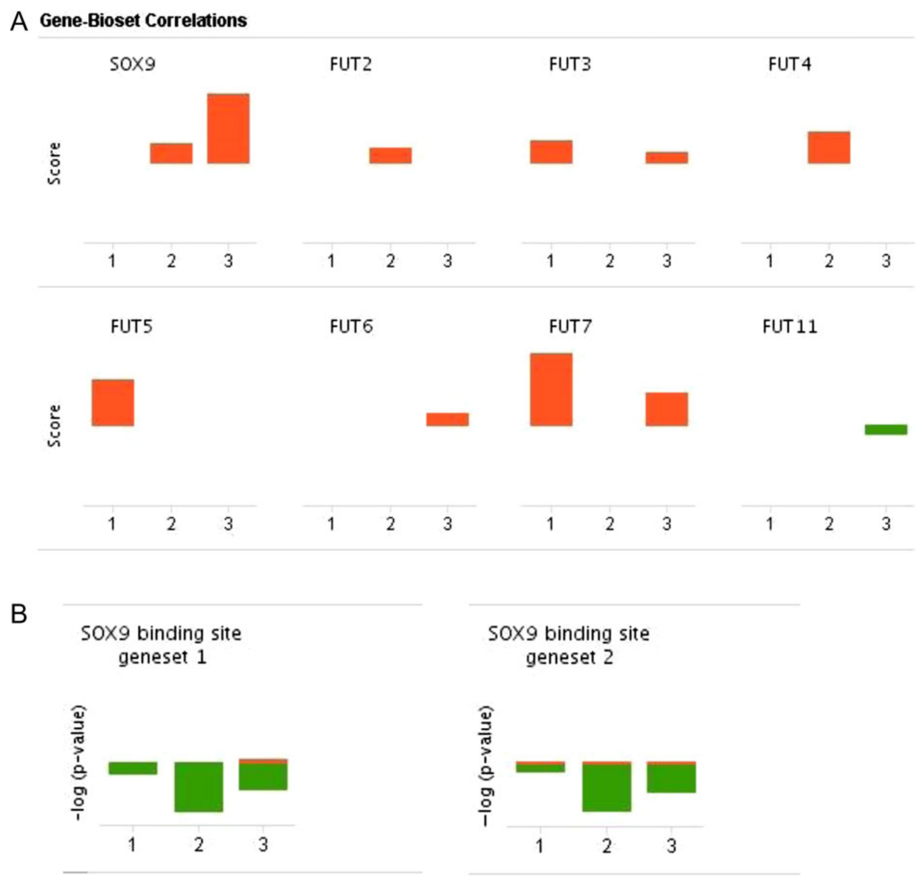

Supplementary Figure S2 The differential expression of (A) all *FUT* genes (that are altered) and *SOX9* genes, and *SOX9* regulated genes from BaseSpace Correlation Engine software (B&C) gene set 1 (*n* = 237 of SOX9 regulated genes; Source Broad Institute's MSigDB. Genes with promoter regions [−2kb,+2 kb] around transcription start site containing the motif NNNNAACAATRGNN which matches annotation for SOX9) and gene set 2 (*n* = 358 of SOX9 regulated genes; Source Broad Institute's MSigDB. Genes with promoter regions [−2kb,+2 kb] around transcription start site containing the motif CATTGTYY which matches annotation for SOX9) was examined in the secretory endometrium of women with endometriosis compared with the same in women without endometriosis in all three publically available microarray datasets of premenopausal endometrial samples from women not on hormonal treatments (*n* = 35; Bioset 1=Endometrium from endometriosis patients at mid-secretory phase vs_ normal (GSE6364, Burney *et al.*, 2007); Bioset 2=Endometria (mid-secretory) - mildly severe endometriosis _vs_ no endometriosis (GSE51981, Tamaresis *et al.*, 2014)) and Bioset 3= Endometria (mid-secretory) – moderately severe endometriosis _vs_ no endometriosis (GSE51981, Tamaresis *et al.*, 2014)) using the BaseSpace Correlation Engine (BSCE; (Kupershmidt *et al.*, 2010) software; https://www.illumina.com/informatics/research/biological-data-interpretation/nextbio.html last accessed on August 8, 2018; Illumina, San Diego, CA, USA). BaseSpace® Correlation Engine is a repository of over 22 000 processed and ontologically tagged omics-scale Studies from the public domain. It uses rank based enrichment statistics to explore hypotheses and validate biomarker and signature activities based on experimental data. Gene-Bioset Correlations display Scores, which are normalised representations of gene significance in each Bioset. Bars represent the absolute Fold Change rank of that gene in a Bioset; Green= down-regulated, red = up-regulated. Supplementary 2A demonstrates the up-regulation of *SOX9* and *FUT2, FUT3, FUT4, FUT5, FUT6* and *FUT7* genes and down regulation of *FUT11* in these three biosets. S2B demonstrates the cumulative and detailed information on differential expression of two sets of potential SOX9 regulated genes included in BaseSpace Correlation Engine software in these three biosets suggesting a functional relevance to the observed SOX9 upregulation. (Supplementary 2C, Supplementary 2D) Individual Bioset-Biogroup Correlation results across SOX9 binding site gene subsets 1 and 2. Each graphic shows the size of Biosets and Biogroups, a Venn Diagram of overlap and Bar Charts with the significance of directional subsets. Significant down regulation of SOX9 controlled genes is evident. **Burney RO, Talbi S, Hamilton AE, Vo KC, Nyegaard M, Nezhat CR, Lessey BA, Giudice LC. Gene expression analysis of endometrium reveals progesterone resistance and candidate susceptibility genes in women with endometriosis. Endocrinology 2007;148:3814–3826. *Tamaresis JS, Irwin JC, Goldfien GA, Rabban JT, Burney RO, Nezhat C, DePaolo LV, Giudice LC. Molecular Classification of Endometriosis and Disease Stage Using High-Dimensional Genomic Data. Endocrinology 2014;155:4986–4999. *Kupershmidt I, Su QJ, Grewal A, Sundaresh S, Halperin I, Flynn J, Shekar M, Wang H, Park J, Cui W et al. Ontology-based meta-analysis of global collections of high-throughput public data. PLoS One 2010;5.*: pii. e13066.

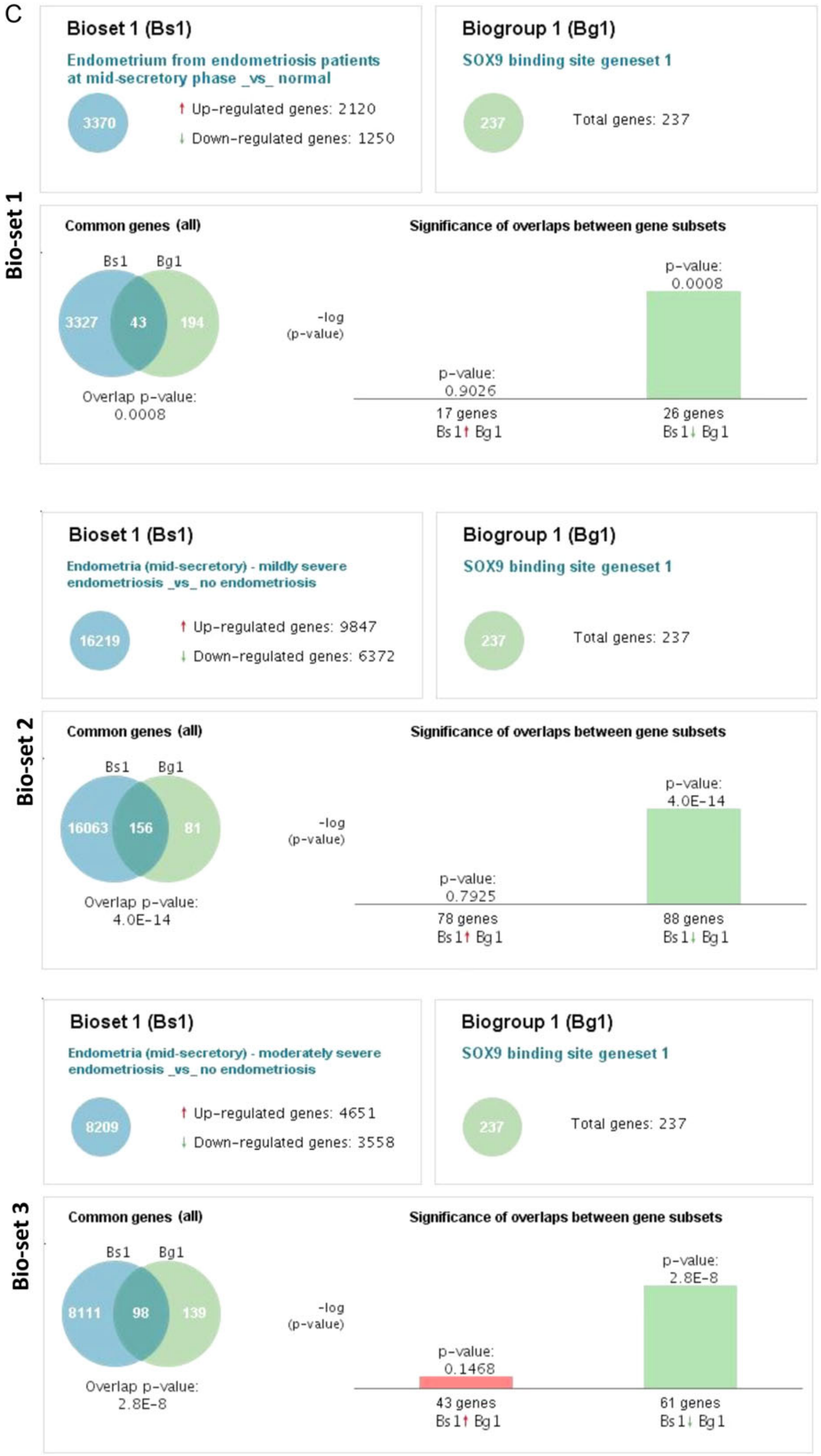

Supplementary Figure S2 *Continued*

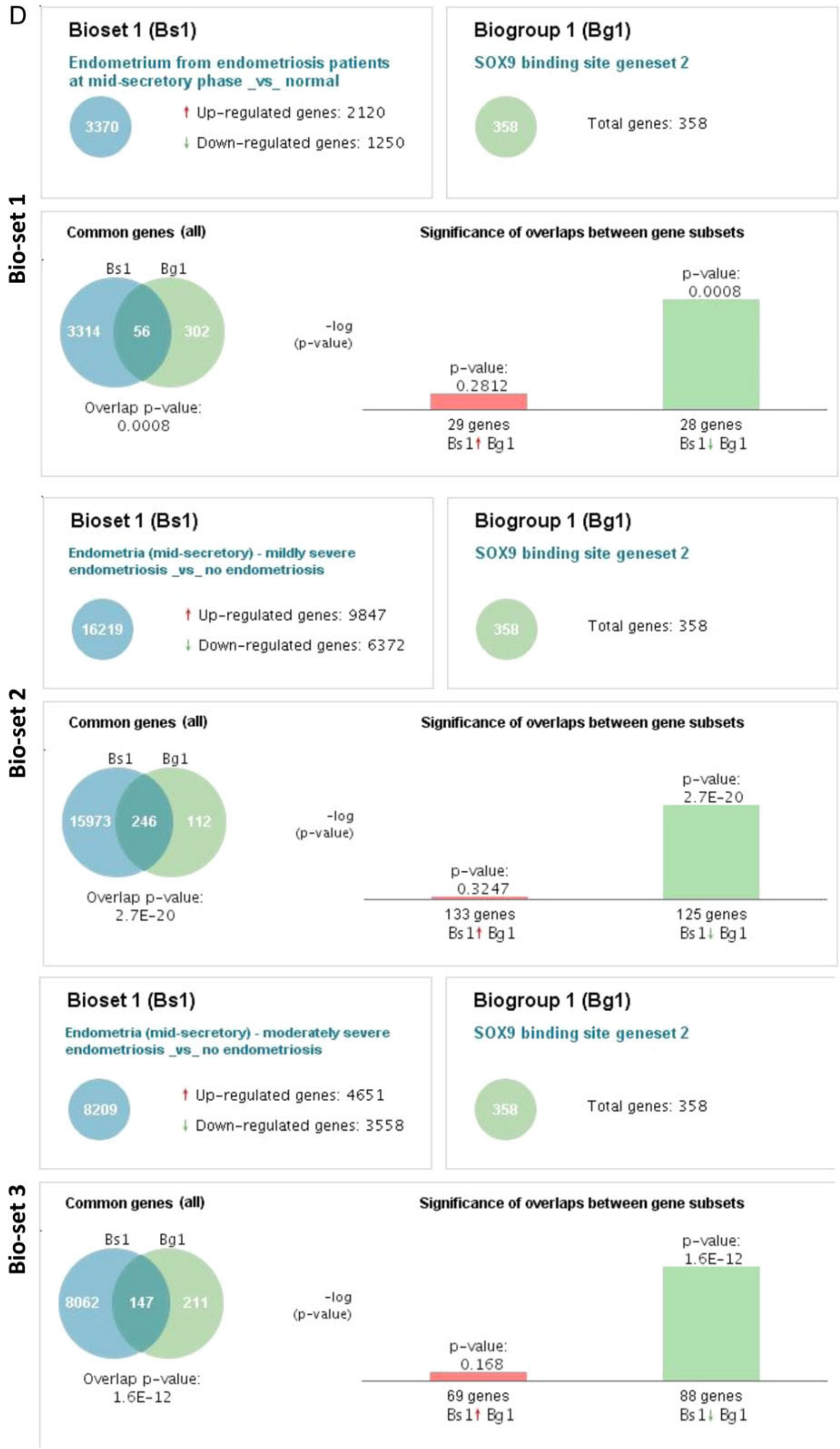

Supplementary Figure S2 *Continued*
