# Supplementary material for: Abnormally located SSEA1+/SOX9+ endometrial epithelial cells with a basalis-like phenotype in the eutopic functionalis layer may play a role in the pathogenesis of endometriosis
Source: Hum Reprod. 2018 Nov 29;34(1):56–68. doi: 10.1093/humrep/dey336 (PMC6295963; doi:10.1093/humrep/dey336)
Supplement: Supplementary Figure 3 [file dey336supplement_figure3.pdf]

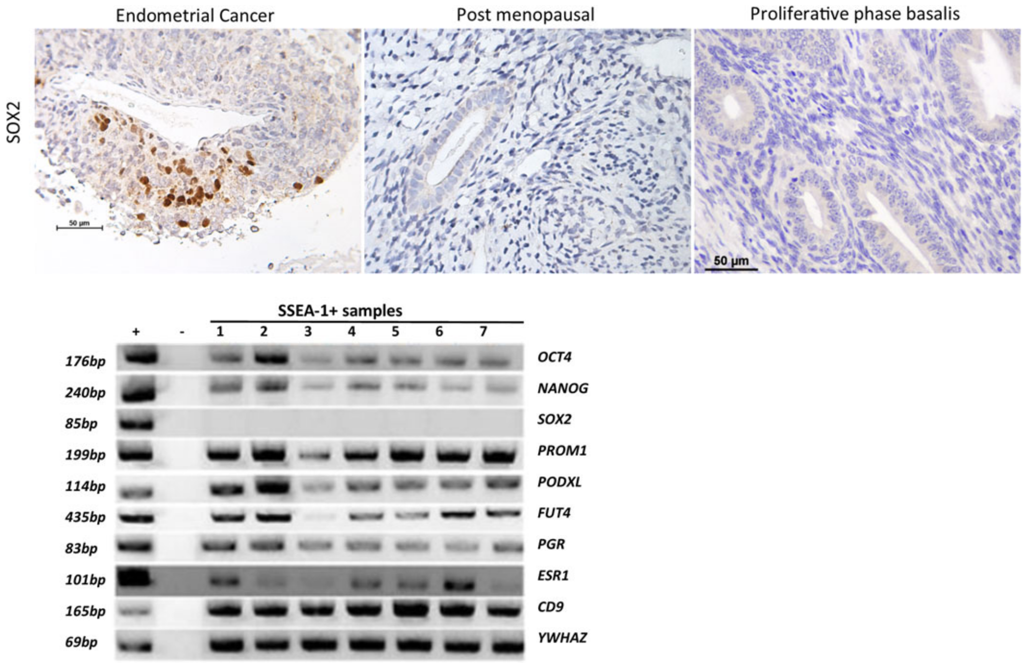

Supplementary Figure S3 Micrographs showing positive nuclear SOX2 expression in endometrial cancer, but no positive staining was observed in the full thickness healthy endometrium. Representative micrographs of postmenopausal endometrium and proliferative phase *basalis* endometrium shown. Agarose gel confirmation of molecular size for PCR amplicons, positive and negative control samples and SSEA1+ sorted cells.
