# Supplementary material for: Abnormally located SSEA1+/SOX9+ endometrial epithelial cells with a basalis-like phenotype in the eutopic functionalis layer may play a role in the pathogenesis of endometriosis
Source: Hum Reprod. 2018 Nov 29;34(1):56–68. doi: 10.1093/humrep/dey336 (PMC6295963; doi:10.1093/humrep/dey336)
Supplement: Supplementary Table 1 [file dey336supplement_table1.pdf]

Supplementary Table SI **Demographic data.**

Subject	Cycle stage	Smoker*	Endometriosis stage	Parity	Age (y)	BMI (kg/m^2)	FT/pipelle
1	Menstrual	0	3–4	0	41	24.2	FT
2	Menstrual	1	2	3	42	22.4	FT
3	Proliferative	0	4	0	39	24.8	pipelle
4	Proliferative	0	2–3	0	38	28	pipelle
5	Proliferative	0	2	0	23	25.5	pipelle
6	Proliferative	0	1	1	36	29.7	pipelle
7	Proliferative	0	1	1	39	25	FT
8	Proliferative	1	2	0	45	32	FT
9	Proliferative	0		1	21	18.7	pipelle
10	Proliferative	0	1	2	41	26.6	pipelle
11	Proliferative	0	1	2	45	27.2	FT
12	Proliferative	0	2	0	31	27	pipelle
13	Secretory	0	4	0	31	20.5	pipelle
14	Secretory	1	1	2	48	22.9	FT
15	Secretory	0	2	0	46	23.9	pipelle
16	Secretory	0	2	0	43	28.4	pipelle
17	Secretory	1	4	0	27	20.6	pipelle
18	Secretory	1	1	0	28	25.5	pipelle
19	Secretory	0	3–4	1	31	28.3	pipelle
20	Secretory	0	3	0	24		pipelle
21	Secretory	0	4	0	46		FT
22	Secretory	0	1	0	38	25.2	pipelle
23	Secretory	0	4	1	39	29.6	FT
24	Secretory	0	4	2	31	26.8	pipelle
25	Secretory	0	2	2	45	20.5	FT
26	Secretory	0	2	2	30	23.8	FT
27	Secretory	0	1	2	33	20.3	FT
28	Secretory	0	1	0	38	23.3	FT
29	Secretory	1	4	1	34	28	FT
30	Secretory	0	2	4	45	29.5	FT
31	Secretory	1	1	3	44	34.9	FT
32	Secretory	0	4	0	48	29.2	FT
33	Secretory	1	1–2	2	41	27.8	pipelle
34	Secretory	1	4	1	37	37.8	pipelle
35	Secretory	0	4	2	27		pipelle
36	Secretory	0	1	0	24	18.4	pipelle
37	Secretory	0	1	0	39	22.8	pipelle
38	Secretory	1	3	0	34	18.9	pipelle
39	Secretory	0	4	1	31	40.6	pipelle
40	Secretory	0	2.5	0	25	17.1	pipelle
41	Secretory	0	4	0	34	35	pipelle
42	Secretory	1	3	1	43	26.2	FT
43	Secretory	1	1	0	29	20.6	pipelle
44	Secretory	0	1		34	26.3	pipelle
45	Menstrual	0	0	3	45	36.4	FT
46	Menstrual	0	0	2	32	26.6	FT
47	Menstrual	0	0	2	38	26.6	pipelle

Continued

Supplementary Table SI *Continued*

Subject	Cycle stage	Smoker*	Endometriosis stage	Parity	Age (y)	BMI (kg/m^2)	FT/pipelle
48	Proliferative	0	0	3	41	24.7	FT
49	Proliferative	1	0	2	45	34.6	FT
50	Proliferative	1	0	2	41	22.6	FT
51	Proliferative	0	0	0	30	25	pipelle
52	Proliferative	1	0	2	32	27.8	FT
53	Proliferative	0	0	3	31	39.8	pipelle
54	Proliferative	0	0	2	42	29.8	pipelle
55	Proliferative	0	0	3	43	40.5	FT
56	Proliferative	0	0	2	45	33.1	FT
57	Proliferative	0	0	1	32	52.2	FT
58	Proliferative	0	0	1	47	23.4	FT
59	Proliferative	0	0	3	46	23.1	FT
60	Proliferative	0	0	2	47	21.6	FT
61	Proliferative	0	0	2	44	38	FT
62	Proliferative	0	0	2	44	31.6	FT
63	Proliferative	0	0	2	37	39.2	FT
64	Proliferative	0	0	1	44	29.6	FT
65	Proliferative	0	0	3	50	26.3	FT
66	Proliferative	1	0	4	47	27.7	FT
67	Proliferative	0	0	1	30	26.7	FT
68	Proliferative	0	0	2	44	24.5	pipelle
69	Proliferative	0	0	1	46	25.4	FT
70	Secretory	0	0	4	41	33.4	FT
71	Secretory	0	0	5	27	23	pipelle
72	Secretory	0	0	2	40	32.4	pipelle
73	Secretory	0	0	3	43	35.2	pipelle
74	Secretory	0	0	4	37	31.6	pipelle
75	Secretory	1	0	2	45	27.4	pipelle
76	Secretory	0	0	3	38	28.1	pipelle
77	Secretory	0	0	5	46	26.8	FT
78	Secretory	1	0	2	46	27.4	FT
79	Secretory	0	0	3	31	24.9	FT
80	Secretory	0	0	2	37	22.7	FT
81	Secretory	1	0	4	41	26.7	FT
82	Secretory	0	0	2	48	37.3	pipelle
83	Secretory	1	0	4	37	21.7	FT
84	Secretory	0	0	3	37	40	FT
85	Secretory	0	0	0	47	22.6	FT
86	Secretory	0	0		45	31.6	FT
87	Secretory	1	0	0	28	24.3	pipelle
88	Secretory	0	0	3	37	41.5	FT
89	Secretory	1	0	2	41	18.9	pipelle
90	Secretory	1	0	6	39	22.4	pipelle
91	Secretory	0	0	2	44	21	pipelle
92	Secretory	0	0	3	30	28.1	FT
93	Secretory	0	0	0	21	25.9	pipelle
94	Secretory	1	0	4	45	21.7	FT

Continued

Supplementary Table SI *Continued*

Subject	Cycle stage	Smoker*	Endometriosis stage	Parity	Age (y)	BMI (kg/m^2)	FT/pipelle
95	Secretory	1	0	2	44	24.3	FT
96	Secretory	1	0	4	39	22.6	FT
97	Secretory	0	0	3	47	19.7	pipelle
98	Secretory	1	0	2	37	26.5	FT
99	Secretory	0	0	4	27	21.1	pipelle
100	Secretory	0	0	2	49	28.4	FT
101	Secretory	0	0	1	45	29.3	FT
102	Secretory	0	0	2	42	30.3	FT

*0 = No 1 = Yes.
FT = full thickness hysterectomy endometrial sample.
