# Supplementary material for: Abnormally located SSEA1+/SOX9+ endometrial epithelial cells with a basalis-like phenotype in the eutopic functionalis layer may play a role in the pathogenesis of endometriosis
Source: Hum Reprod. 2018 Nov 29;34(1):56–68. doi: 10.1093/humrep/dey336 (PMC6295963; doi:10.1093/humrep/dey336)
Supplement: Supplementary Table 2 [file dey336supplement_table2.pdf]

Supplementary Table SII **Antibody details.**

Target	Host species	Clone	Antigen retrieval conditions	Dilution	Incubation time & temperature	Supplier
SSEA1	Mouse	MC-480	Tris-EDTA pH 9.0	1:800	4°C overnight	Biolegend, London, UK
SOX2	Rabbit	D6D9	Citrate pH 6.0	1:50	4°C overnight	Cell Signalling Technology, Herts, UK
SOX9	Goat	N/A	Citrate pH 6.0	1:400	4°C overnight	Cell Signalling Technology, Herts, UK
Ki67	Mouse	MM1	Citrate pH 6.0	1:200	4°C overnight	Leica (Novocastra) Newcastle, UK
ERα	Rabbit	N/A	Citrate pH 6.0	1:50	Room temperature, 2 h	Abcam, Cambridge, UK
ERβ	Mouse	PPG5/10	Citrate pH 6.0	1:50	4°C overnight	Biorad (AbD Serotec) Kidlington, UK
PR	Mouse	PgR636	Citrate pH 6.0	1:1000	Room temperature, 30 min	DAKO (Agilent), Stockport, UK
Laminin	Rabbit	N/A	Proteinase K	1:800	4°C overnight	DAKO (Agilent), Stockport, UK
Cytokeratin 18	Mouse	DC10	Citrate pH 6.0	1:800	4°C overnight	DAKO (Agilent), Stockport, UK
β-catenin	Rabbit	6B3	Citrate pH 6.0	1:200	4°C overnight	Cell Signalling Technology, Herts, UK
AR	Mouse	AF-441	Citrate pH 6.0	1:75	4°C overnight	DAKO (Agilent), Stockport, UK
MUC-1	Mouse	BC2	Citrate pH 6.0	1:100	4°C overnight	Biorad (AbD Serotec) Kidlington, UK

Supplementary Table SIII **Primers used for qPCR.**

Gene	qPCR primer sequence	Product Size (bp)	Annealing Temperature (°C)	Source
OCT4	F: 5′AGAACCGAGTGAGAGGCAA3′<br>R: 5′CTCTCGTTGTGCATAGTCGC3′	176	56	In house
NANOG	F: 5′CGGAGACTGTCTCTCCTCTT3′<br>R: 5′GTTCTTGCATCTGCTGGAGG3′	240	56	In house
SOX2	F: 5′CGAGATAAACATGGCAATCAAAAT3′<br>R: 5′AATTCAGCAAGAAGCCTCTCCTT3′	85	56	(Wong *et al.*, 2010)
PODXL	F: 5′CCATCGTCTGCATGGCATCA3′<br>R: 5′CTGTCTGCAGCTCCTCTGTT3′	114	56	In house
CD133	F: 5′TGCAACAGCATCAGATTGTC3′<br>R: 5′TACCTGCTACGACAGTCGTG3′	199	56	In house
CD9	F: 5′GACACCTACAACAAGCTGAA3′<br>R: 5′ACAGGACTTCACGGTGAAGG3′	165	56	In house
ERα	F: 5′TGATTGGTCTCGTCTGGCG3′<br>R: 5′CATGCCCTCTACACATTTTCCC3′	101	56	(Henderson *et al.*, 2003)
PR	F: 5′CAGTGGGCGTTCCAAATGA3′<br>R: 5′TGGTGGAATCAACTGTATGTCTTGA3′	83	56	(Henderson *et al.*, 2003)
FUT4	F: 5′CAGCTGGTTCGAGCGGTGAAGCCGCGCT3′<br>R: 5′CAGAAAAACGTGAATCGGGAACAGTTGTGT3′	435	60	(Ponnampalam *et al.*, 2008)
FUT3	F: 5′GCCGACCGCAAGGTGTAC3′<br>R: 5′TGACTTAGGGTTGGACATGATATCC3′	75	60	(Higai *et al.*, 2006)
SOX9	F: 5′ GTACCCGCACTTGCACAAC 3′<br>R: 5′ TCTCGCTCTCGTTCAGAAGTC 3′	74	61	(Masuda *et al.*, 2010)
YWHAZ	F: 5′CGTTACTTGGCTGAGGTTGCC3′<br>R: 5′GTATGCTTGTTGTGACTGATCGAC3′	69	56	(Marullo *et al.*, 2010)

References

Henderson TA, Saunders PT, Moffett-King A, Groome NP, Critchley HO. Steroid receptor expression in uterine natural killer cells. *The Journal of Clinical Endocrinology and Metabolism* 2003;88:440–449.

Higai K, Ishihara S, Matsumoto K. NFkappaB-p65 dependent transcriptional regulation of glycosyltransferases in human colon adenocarcinoma HT-29 by stimulation with tumour necrosis factor alpha. *Biological & Pharmaceutical Bulletin* 2006;29:2372–2377.

Marullo M, Zuccato C, Mariotti C, Lahiri N, Tabrizi SJ, Di Donato S, Cattaneo E. Expressed Alu repeats as a novel, reliable tool for normalisation of real-time quantitative RT-PCR data. *Genome Biol* 2010;11.

Masuda H, Matsuzaki Y, Hiratsu E, Ono M, Nagashima T, Kajitani T, Arase T, Oda H, Uchida H, Asada H *et al.* Stem cell-like properties of the endometrial side population: implication in endometrial regeneration. *PLoS One* 2010;5: e10387.

Ponnampalam AP, Gargett CE, Rogers PA. Identification and hormonal regulation of a novel form of NKp30 in human endometrial epithelium. *Eur J Immunol* 2008;38:216–226.

Wong OG, Huo Z, Siu MK, Zhang H, Jiang L, Wong ES, Cheung AN. Hypermethylation of SOX2 Promoter in Endometrial Carcinogenesis. *Obstet Gynecol Int* 2010;2010.
